# A Long-Day Photoperiod and 6-Benzyladenine Promote Runner Formation through Upregulation of Soluble Sugar Content in Strawberry

**DOI:** 10.3390/ijms21144917

**Published:** 2020-07-12

**Authors:** Yali Li, Jiangtao Hu, Hao Wei, Byoung Ryong Jeong

**Affiliations:** 1 Division of Applied Life Science (BK21 Plus Program), Department of Horticulture, Graduate School of Gyeongsang National University, Jinju 52828, Korea; leeyali@gnu.ac.kr (Y.L.); hujiangtao@gnu.ac.kr (J.H.); oahiew@gmail.com (H.W.); 2 Institute of Agriculture and Life Science, Gyeongsang National University, Jinju 52828, Korea; 3 Research Institute of Life Science, Gyeongsang National University, Jinju 52828, Korea

**Keywords:** orthogonal test, photoperiod, DIF, plant growth regulator, strawberry propagation, sugar

## Abstract

Commercial strawberries are mainly propagated using daughter plants produced on aerial runners because asexual propagation is faster than seed propagation, and daughter plants retain the characteristics of the mother plant. This study was conducted to investigate the effective factors for runner induction, as well as the molecular mechanisms behind the runner induction. An orthogonal test with 4 factors (photoperiod, temperature, gibberellin, and 6-benzyladenine), each with 3 levels was performed. Proteins were also extracted from the crowns with or without runners and separated by two-dimensional electrophoresis. The results of the orthogonal test showed that a long-day (LD) environment was the most influential factor for the runner formation, and 50 mg·L^−1^ of 6-BA significantly increased the number of runners. A proteomic analysis revealed that 32 proteins were differentially expressed (2-fold, *p* < 0.05) in the strawberry crowns with and without runners. A total of 16 spots were up-regulated in the crowns with runners induced by LD treatment. Identified proteins were classified into seven groups according to their biological roles. The most prominent groups were carbohydrate metabolism and photosynthesis, which indicated that the carbohydrate content may increase during runner formation. A further analysis demonstrated that the soluble sugar content was positively correlated with the number of runners. Thus, it is suggested that the photoperiod and 6-BA break the dormancy of the axillary buds and produce runners by increasing the soluble sugar content in strawberry.

## 1. Introduction

Cultivated strawberry (*Fragaria × ananassa*) is one of the most popular fruits all around the world due to its beautiful appearance, flavor and health benefits. The cultivation and production of strawberry has continuously increased over the last two decades [1]. Commercially, strawberries are mainly propagated using daughter plants produced on runners, because asexual production using daughter plants is faster than seed propagation, and daughter plants retain the characteristics of the mother plant [2]. Hence, the ability to form runners carries an enormous agricultural importance. Strawberry is a rosette-forming plant, with short stems that are also called the primary crown. At the base of each leaflet along the primary crown, there is an axillary meristem (AXM), and these AXMs can either develop into runners and branch crowns, or remain dormant [3]. A high number of branch crowns means more inflorescence because only the shoot apical meristem (SAM) can develop an inflorescence [4]. It has long been observed that wild and cultivated strawberry have antagonistic processes of developing runners and flowers from different meristem crowns. Therefore, the development of the AXM is extremely important for runner production.

Factors affecting runner induction in strawberry have been studied for several decades. Seasonal flowering (June-bearing or non-remontant) and perpetual flowering (everbearing or remontant) are two different genotypes of commercial strawberries [5,6,7]. It is clear that long-days (LD) and higher temperatures promote runner formation in the seasonal flowering strawberries [8,9,10]. While higher temperatures also increase the runner production in the perpetual flowering strawberries, the effects of the photoperiod on the runner production in the perpetual flowering strawberries vary among different experiments [11,12]. Furthermore, plant growth regulators (PGRs) also play a decisive role in the fate of the AXMs. The most well-known PGRs for runner induction are gibberellins (GA). Numerous studies have shown that GA can significantly increase the runners in both wild and cultivated strawberries [13,14,15]. GA biosynthesis inhibitor prohexadione-calcium (Pro-Ca) retards the runner formation [4,16,17]. In addition, cytokinine and auxin coordinate the dormancy and outgrowth of the axillary buds in strawberry [18], and exogenous applications of benzyladenine (BA) were observed to contribute to a higher number of runners in some experiments [19,20].

Researchers began to focus on the genetic factors affecting the runner formation in strawberry in recent years. Diploid woodland strawberry (*Fragaria vesca*) has been developed as a model for the commercial strawberry (octoploid) because of the small genome size [21]. Tenreira and his colleagues [22] found that the runnerless trait in woodland strawberry was caused by a deletion in the active site of a GA biosynthesis gene named *FveGA20ox*. This mutation was also found in all natural runnerless woodland strawberry plants, and exogenous applications of bioactive GA restored the runner-producing phenotype in the runnerless plants. Later, *FveRGA1* was identified as a key gene controlling the runner formation in woodland strawberry plants. FveRGA1 belongs to DELLA proteins, which function as a GA signaling suppressor, and silencing *FveRGA1* results in constitutive GA responses and constant development of runners [2,23]. Moreover, Mouhu et al. [10] suggested that *FvSOC1* plays a central role in the photoperiodic control of runner production and flowering in woodland strawberries, and this process may also be regulated through the activation of GA biosynthesis genes. These results strongly suggest that GA biosynthesis in the axillary meristem is essential for inducing runner differentiation in diploid strawberry plants [24]. Few studies report on the genetic control of octoploid cultivated strawberry. Some studies have shown that perpetual flowering is controlled by a single dominant gene in cultivated strawberry plants [25,26], but the relation of these genes and runner production has not yet been identified. More studies have favored the polygenic control of the runnering trait. Gaston et al. [27] found that a single major quantitative trait locus (QTL) named *FaPFRU* negatively regulates the runnering in cultivated strawberry, which was further confirmed by Sooriyapathirana et al. [28]. Moreover, Hossain et al. [1] identified seven other QTLs, namely *qRU-5D*, *qRU-3D1*, *qRU-1D2*, *qRU-4D*, *qRU-4C*, *qRU-5C*, and *qRU-2D2*, that are responsible for the runner formation. These QTLs are not orthologous to the loci affecting runnering in *Fragaria vesca*, suggesting that runnering in diploid and octoploid strawberry are controlled by different genes. Although great progress has been made to reveal the genetic controls underlying the runner formation in strawberry, the proteomic changes underlying strawberry runnering are poorly understood. Proteomics is a reliable approach in understanding biological processes. Analyzing the proteomic changes during runner formation may help us understand the inner mechanisms.

In this study, we compared the effects of the photoperiod, temperature, GA_3_ and 6-BA on the runner induction in one of the most commonly cultivated strawberries in South Korea. We also compared the proteome-level changes in the crowns of the strawberry with and without runners. These results will provide new insights for the propagation of cultivated strawberry.

## 2. Results

### 2.1. Analysis of the Morphological and Growth Parameters

**Figure 1:**
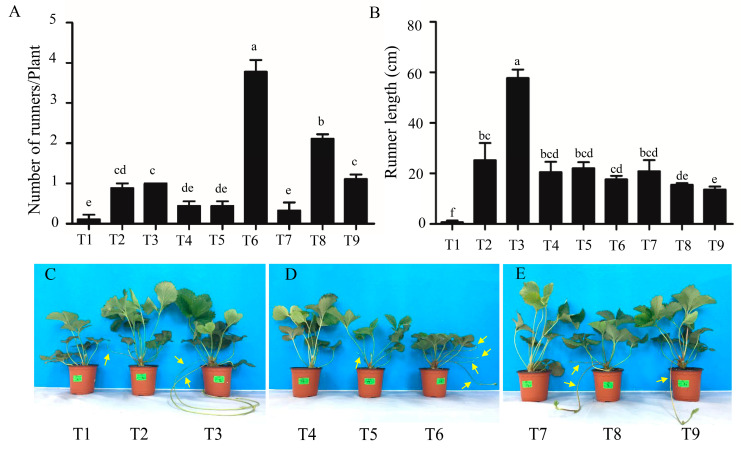
The morphology of the strawberry plants affected by the photoperiod, temperature, gibberellin (GA_3_) and 6-benzyladenine (6-BA). (**A**) Number of runners per plant; (**B**) runner length; (**C**–**E**) present the morphology of the strawberry plants in T1 to T9, respectively. Runners are indicated with yellow arrows. T1, 23/27 °C + 8/16 h; T2, 23/27 °C + 12/12 h + 50 mg·L^−1^ GA_3_ + 50 mg·L^−1^ BA; T3, 23/27 °C + 16/8 h + 100 mg·L^−1^ GA_3_ + 100 mg·L^−1^ BA; T4, 25/25 °C + 8/16 h + 50 mg·L^−1^ GA_3_ + 100 mg·L^−1^ BA; T5, 25/25 °C + 12/12 h + 100 mg·L^−1^ GA_3_; T6, 25/25 °C + 16/8 h + 50 mg·L^−1^ BA; T7, 27/13 °C + 8/16 h + 100 mg·L^−1^ GA_3_ + 50 mg·L^−1^ BA; T8, 27/13 °C + 12/12 h + 100 mg·L^−1^ BA; T9, 27/13 °C + 16/8 h + 50 mg·L^−1^ GA_3_. The plants used for this experiment were one month and a half after cutting propagation and pictures were taken after another month cultivation in growth chambers. Data are presented as the mean ± standard error (*n* = 3). Lowercase letters indicate the significant difference according to Duncan’s multiple range test at a 0.05 level.

**Figure 2:**
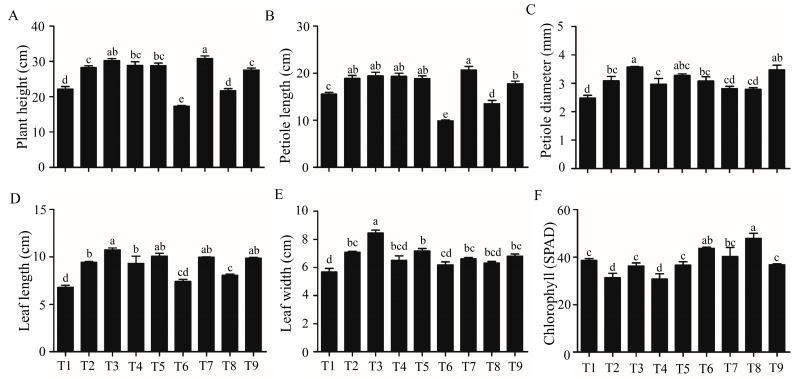
The growth parameters and chlorophyll content for each treatment. (**A**) Plant height; (**B**) petiole length; (**C**) petiole diameter; (**D**) leaf length; (**E**) leaf width; (**F**) chlorophyll. Data are presented as the mean ± standard error (*n* = 3). Lowercase letters indicate the significant difference according to Duncan’s multiple range test at a 0.05 level.

After 4 weeks of cultivation in three plant growth chambers, different morphologies were observed in strawberry plants (Figure 1C–E). The highest number of runners induced per plant was found in T6, and then T8 (Figure 1A). The longest runner was found in T3 (Figure 1B). The range analysis revealed that the photoperiod was the most important factor for runner induction; the long-day (LD) photoperiod (16 h) led to a strong increase in the number of runners (Table 1). The optimal day/night temperature for runner formation was 25/15 °C. 6-BA also promoted the runner induction, where a concentration of 50 mg·L^−1^ was the most effective. Foliar GA_3_ spray decreased the number of runners, where the concentration and the number of runners were negatively correlated. The plant height and petiole length were the shortest in T6 (Figure 2A,B), and the leaf length was also shorter in T6 compared to that of plants treated with GA_3_ (Figure 2D). The chlorophyll content (SPAD) was also higher in T6 and T8 (Figure 2F). Furthermore, through the range analysis, it was found that GA_3_ significantly increased the runner length, plant height, petiole length, petiole diameter, leaf length, and leaf width, but decreased the leaf chlorophyll content (SPAD), while a high daytime temperature increased the chlorophyll content (Tables S1–S7).

**Figure 3:**
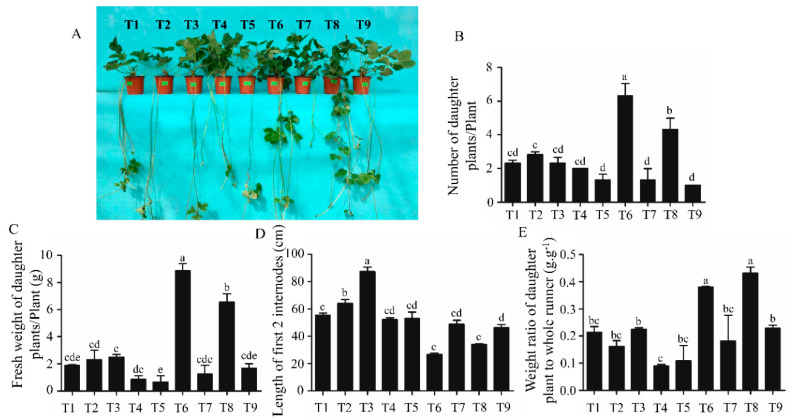
The morphological and growth parameters of the strawberry plants after a 5-week cultivation in a glasshouse. (**A**) Morphology of the strawberry plants; (**B**) number of daughter plants per plant; (**C**) fresh weight of daughter plants; (**D**) length of the first 2 internodes; (**E**) weight ratio of the daughter plant to the whole runner. Data are presented as the mean ± standard error (*n* = 3). Lowercase letters indicate the significant difference according to Duncan’s multiple range test at a 0.05 level.

Half of the strawberry plants were moved to a glasshouse after cultivation in growth chambers. The data related to runners were collected 5 weeks later. T6 produced the highest number of daughter plants and also resulted in the greatest fresh weight of the daughter plants per plant. T8 produced the second highest number of daughter plants, with the second highest average daughter plant fresh weight (Figure 3B,C). On the contrary, the length of the first two internodes was shorter for plants in T6 and T8 (Figure 3A,D). The weight ratio of the daughter plant to the whole runner was also significantly increased in T6 and T8 when compared that in the other treatments (Figure 3E).

### 2.2. Protein Expression Profiles and Protein Identification

**Figure 4:**
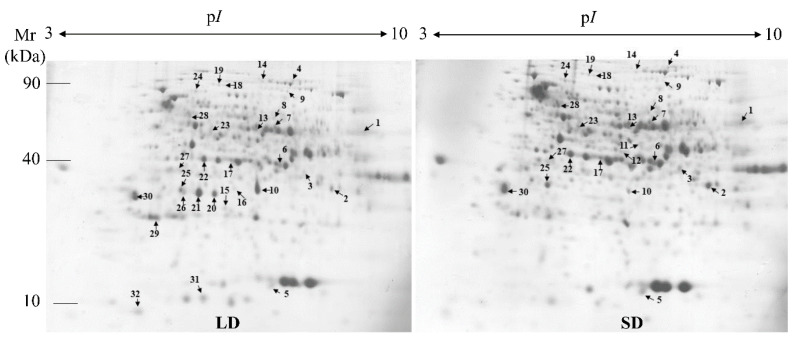
Representative 2-DE gel maps of proteins in the crowns of strawberry plants grown with strawberry plants with runners (LD) and without runners (SD) photoperiods. The 2-DE were replicated three times and the significant differences were calculated by Student’s *t*-test. Numbers and arrows indicate the differentially expressed protein (DEP) spots with at least 2-fold changes between the crown with and without runners.

**Figure 5:**
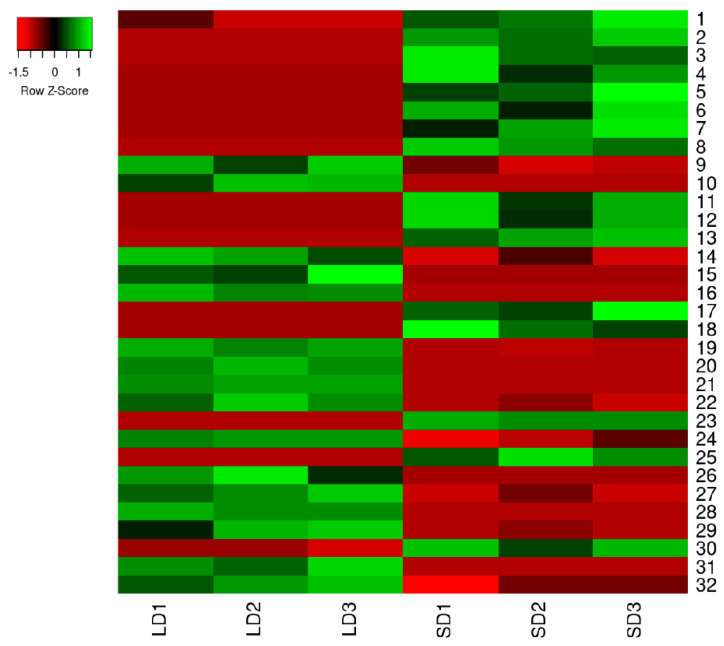
The expression profiles of 32 DEPs under LD and SD conditions. LD1, LD2, LD3, SD1, SD2, and SD3 represent the three replicates in each treatment. The significant differences were calculated by Student’s *t*-test.

The proteins in the crowns of the strawberry plants with runners (LD) and without runners (SD) were separated according to their M_r_ and p*I* (Figure 4). Approximately 657 protein spots were detected in all replicates. Comparative proteomic analyses revealed that 32 spots showed significant and reproducible differences in abundance (*p* < 0.05 and at least 2-fold) in the two different types of crowns. A total of 16 spots (spots 9, 10, 14, 15, 16, 19, 20, 21, 22, 24, 26, 27, 28, 29, 31, and 32) were up-regulated in the crowns with runners when compared to those without crowns (Figure 5). Among these, eight spots (spots 15, 16, 20, 21, 26, 29, 31, and 32) were detected only in crowns with runners, and two spots (spots 11 and 12) were detected only in runnerless crowns. The differentially expressed protein (DEP) spots were identified by the MALDI-TOF/MS analysis, and 25 spots were successfully identified (Table 2). Most of the identified proteins were matched to proteins in the *Fragaria vesca* database.

### 2.3. Functional Classification of the DEPs

The identified proteins were classified into seven groups (Table 2). The most prominent groups were carbohydrate metabolism- and photosynthesis-related proteins, followed by proteins related to proteometabolism, stress, signaling, general metabolism, and respiration. Spots 3, 8, 9, 20, and 25 were involved in carbohydrate metabolism. Among these, spots 9 and 20 were significantly increased, while others decreased, in the crown with runners. Four photosynthesis-related spots (14, 19, 24, and 28) increased, while one photosynthesis-related protein (spot 13) decreased, in the crown with runners. Spots 6, 7, 12, and 23 were assigned to proteometabolism, and these proteins decreased in the crown with runners. There are four stress-related proteins identified: two of them (spots 1 and 4) increased in the crown without runners, while the other two (spots 31 and 32) were expressed more in the crown with runners. Moreover, one signaling protein (spot 16) was highly expressed, while two signaling proteins (spot 2 and 5) decreased, in the crown with runners. In total, three general metabolism-related proteins (spots 11, 17 and 29) were found to be differently expressed in the crown with vs. without runners, all of them except for spot 29 decreasing in the crown with runners. Furthermore, one respiration-related protein (spot 10) was highly expressed in the crown with runners.

### 2.4. Soluble Sugar and Starch Contents

**Figure 6:**
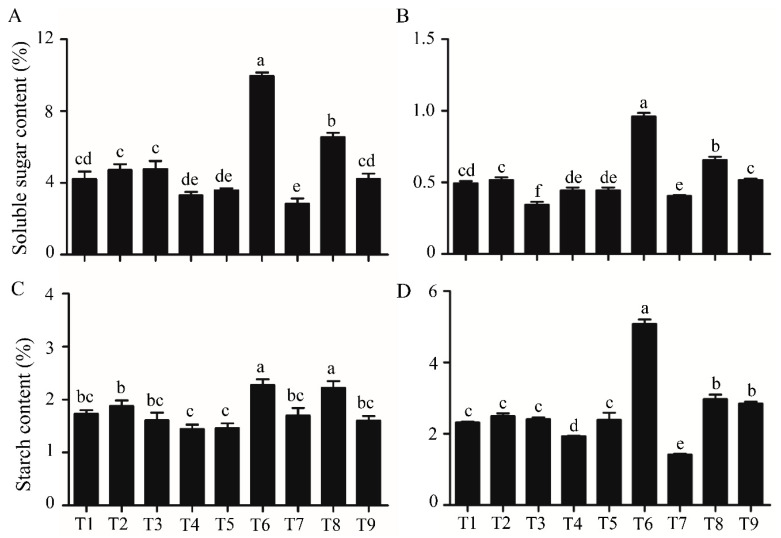
The sugar and starch contents in strawberry plants treated with different photoperiods, temperatures, GA_3_ and 6-BA concentrations. (**A**,**B**) Soluble sugar contents in young leaves and crowns, respectively. (**C**,**D**) Starch contents in young leaves and crowns, respectively. Data are presented as the mean ± standard error (*n* = 3). Lowercase letters indicate the significant difference according to Duncan’s multiple range test at a 0.05 level.

The soluble sugar contents in young leaves and crowns were significantly increased in T6 and T8 when compared to those in the other treatments (Figure 6A,B). The sugar content in leaves of strawberry plants grown in T6 was more than twice that of plants grown in the other treatments, except for T8. The starch contents were also increased in young leaves and crowns in T6, especially for the content of starch in crowns (Figure 6C,D). Range analysis revealed that photoperiod and 50 mg·L^−1^ 6-BA increased while GA_3_ decreased sugar and starch contents (Tables S8–S11).

## 3. Discussion

Because runner formation is photoperiod- and temperature- sensitive, we compared the impact of the photoperiod, temperature, GA_3_ and 6-BA on the runner induction in the strawberry ‘Seolhyang’. The results showed that the photoperiod was the most influential factor for runner induction, and the LD condition significantly increased the number of runners compared to the SD, which is consistent with the findings from previous studies [12,29]. Higher temperatures proved to be crucial for runner induction [30], so three different sets of day and night temperatures (DIF) with the same mean daily temperature of 20 °C were used in this study. The DIF of 25/15℃ was the most optimal for runner induction, indicating that both day and night temperatures are important for runner formation. The effects of the BA concentration on runner formation still remain controversial, while some researchers found that BA could improve runner induction [20], others have shown that BA alone had no effects on producing runners [31,32]. Our results showed that 6-BA (T6 and T8) significantly increased the number of runners. A recent research revealed that auxin and cytokinin coordinate the dormancy and outgrowth of axillary buds in strawberry. Relatively higher auxin activity is present in the dormant buds, and increased cytokinin activity is present in the non-dormant buds. Both the reduction in auxin accumulation and exogenous cytokinin application could trigger the regeneration of vegetative shoots in dormant buds [18]. Thus, the outgrowth of the axillary buds is affected not only by the cytokinin content, but also by auxin content. GA has been shown to play a critical role in runner formation in woodland strawberry. In this study, exogenous GA_3_ application decreased the number of runners. Surapornpiboon [33] also found that the runner production was negatively correlated with the GA_3_ concentration in cultivated strawberry, which is consistent with our results. Cultivated strawberries are complex allo-octoploids, with four relatively similar sub-genomic chromosome sets from diploid donors. A polyploid usually produces more hormones, total proteins, sugars, flavonoids, etc., compared to the corresponding diploid [34,35,36]. It could be deduced that the octoploids could increase the level of GAs, and these GAs alone are enough for producing runners in ‘Seolhyang’. Thus, the GA_3_ application broke the dynamic equilibrium and reduced the runner formation. In addition, the lengths of the first two internodes decreased while the weight ratios of the daughter plant to the whole runner increased in T6 and T8, which suggested that less energy was wasted.

Functional classification of the DEPs suggested that the carbohydrate content was quite different between strawberry plants with runners and those without runners. Sucrose synthase 2 (spot 9) and glucan endo-1,3-beta-glucosidase (spot 20) were found highly expressed in the crown with runners. The sucrose synthase (SUS) has been associated with the synthesis of both storage and structural carbohydrates. It catalyzes the reversible conversion of sucrose and the UDP to fructose and the UDP-glucose [37]. The expression level of the SUS has been found highly correlated with phloem loading, which is the starting point for exporting process of carbohydrates and other nutrients from leaves [38]. Overexpression of the SUS leads to higher concentrations of fructose in leaves, whereas in elongating fibers, concentrations both glucose and fructose increased [39]. Glucan endo-1,3-beta-glucosidase is mainly present in vacuoles and plays a key role in carbohydrate metabolism [40]. It has the capacity to break the C-N bond and produce oligosaccharides [41]. Thus, the higher expression levels of the SUS and glucan endo-1,3-beta-glucosidase may suggest that more sugars were present in the crown with as opposed to without runners. The expression of malate dehydrogenase (MDH), glucose-6-phosphate 1-dehydrogenase (G6PDH1), and lactoylglutathione lyase increased in the crown without runners. The MDH catalyzes the interconversion of malate and oxaloacetate [42]. It promoted glycolysis in actively proliferating cells [43] and may impair photosynthesis, since promoted photosynthesis was found in tomato (*Solanum lycopersicum*) plants when the activity of the MDH decreased [44]. The G6PDH1 is a key regulatory enzyme in the oxidative pentose phosphate pathway that plays a central role in plant metabolism by converting glucose to ribose-5-phosphate for the biosynthetic processes such as assimilation of nitrogen and fatty-acid [45]. Lactoylglutathione lyase is also related to glycolysis [46]. Therefore, all these enzymes are involved in sugar consumption, which indicates less sugar content in the crown without as opposed to with runners.

Four proteometabolism-related proteins increased in the crown without runners. Protein disulfide-isomerase and mitochondrial-processing peptidase are involved in processing of proteins. Fumarylacetoacetase is an important enzyme involved in the amino acid catabolism [47], and the 26S protease regulatory subunit participates in protein degradation [48]. All these proteins were highly expressed in the crown without runners, which indicates that proteometabolism was enhanced under the SD condition.

Both spots 13 and 24 belong to the ribulose-1,5-bisphosphate carboxylase (RuBisCo) large subunit. RuBisCo is a key enzyme for photosynthesis. It mediates the fixation of atmospheric CO_2_ in the Calvin cycle to produce organic carbon [49]. However, RuBisCo struggles in distinguishing CO_2_ from O_2_. The fixation of O_2_ results in energetically wasteful photorespiration [50]. In this study, spots 13 and 24 were presumed to be involved in photorespiration and photosynthesis, respectively, and spot 24 was more highly expressed under the LD condition, while spot 13 was less highly expressed. Moreover, two ATP-dependent photosynthesis-related chaperone proteins ClpC (spot 19) and Cpn60-2 (spot 28), together with magnesium ion binding protein (spot 14), were increased under the LD condition. ClpC and Cpn60 played an essential role in the photosynthetic activity and photosystem content [51,52,53]. Magnesium, the central element in chlorophyll [54], could affect the RuBisCo activity and is extremely crucial for photosynthesis [55]. These results indicate that photosynthesis was enhanced under the LD condition, which suggests that a greater amount of carbohydrates (such as sugar and starch) was produced by photosynthesis.

The enhanced respiration may also play an important role in the runner formation process. CoQ10 is a conserved CoQ-binding protein that is essential for proper respiration [56]. The expression level of CoQ10 (spot 10) was significantly increased under the LD condition (*p* < 0.05), which suggested that the respiration might be enhanced. Respiration is the metabolic bridge from photosynthesis to growth, where photosynthates are converted into substances that contribute to the plant growth, maintenance, transport, and nutrient assimilation processes [57]. Respiration achieves this process by breaking down sugars into smaller molecules (carbon skeleton intermediates) for biosynthesis, and generating ATP for all heterotrophic energy-requiring processes [58]. The enhanced photosynthesis and respiration under the LD condition indicate that more substances and energy were produced to support plant growth. Since runner formation was the most conspicuous growth difference between plants grown in the LD and SD conditions, we assume that most of the substances and energy produced by respiration were used for producing runners. However, the exact mechanisms with which carbon skeleton intermediates and energy promote runner formation are still an open question.

Recent research has proven that sugar is crucial for the bud outgrowth. For almost a century, the plant growth regulator auxin has been central to theories on apical dominance, whereby the growing shoot tip suppresses the growth of the axillary buds below. Actually, auxin transports very slowly, typically at 1 cm·h^−1^ through the stem [59], but it has been observed that pea (*Pisum sativum*) buds release up to 24 h before changes in the auxin content were observed in the adjacent stem. After the loss of the shoot tip, sugars are rapidly redistributed over large distances (150 cm·h^−1^) and accumulate in axillary buds within a timeframe that correlates with the bud release. Thus, a new theory of apical dominance was put forward that the shoot tip′s strong demand for sugars inhibits axillary bud outgrowth by limiting the amount of sugar translocated to those buds [60]. Furthermore, recent studies have shown that sugar not only plays a nutritional role, but also serves as a significant signaling mediator for bud release. For instance, sugar could suppress the auxin-induced strigolactone pathway to promote bud growth in rose (*Rosa hybrida* L. cv Radrazz) [61]. Moreover, higher expression levels of sugar metabolism and signaling genes were detected in non-dormant buds in strawberry runners when compared with the dormant buds [18]. Thus, we measured the soluble sugar and starch contents in strawberry treated with different photoperiods, temperatures, GA_3_ and 6-BA concentrations. Although T6 and T8 had different photoperiods, they both processed higher soluble sugar and starch contents than the other treatments, and the soluble sugar content was positively correlated with the number of runners, suggesting that sugar may play an important role in breaking the dormancy of the axillary buds and producing runners in strawberry. In addition, a range analysis revealed that GA_3_ significantly decreased the chlorophyll and soluble sugar contents in strawberry. These may be the means for the downregulation of the number of runners by GA_3_ in this experiment (Tables S7–S9). The range analysis also indicated that 6-BA slightly increased the sugar content (Tables S8 and S9). There is evidence demonstrating that 6-BA could promote photosynthesis and increase the soluble sugar content in plants [62,63], which suggests that 6-BA may promote runner formation partly by increasing the sugar content.

## 4. Materials and Methods

### 4.1. Plant Materials and Culture Condition 

The runner plants of the strawberry ‘Seolhyang’ were obtained from a strawberry farm (Sugok-myeon, Jinju, Gyeongsangnam-do, Korea) and were stuck in the BVB Medium (Bas Van Buuren Substrate, EN-12580, De Lier, Westland, The Netherlands) in 21-cell zigzag trays (21-Zigpot/21 cell tray, Daeseung, Jeonju, Korea). All cuttings were kept on a fogged propagation bench with an 80% relative humidity for 2 weeks, and were subsequently cultivated for a month in a glasshouse with 29/20 °C day/night temperatures, an average light intensity of 450 μmol·m^−2^·s^−1^ PPFD coming from the sun, and a natural photoperiod of 12 h. The strawberry plants were transplanted into 10-cm plastic pots for the subsequent experiments.

An orthogonal design L_9_ (3)^4^ was used, where the 9 treatments contained different combinations of photoperiod (8, 12, or 16 h), temperature (23/17, 25/15, or 27/13 °C for day/night), GA_3_ (0, 50, or 100 mg·L^−1^), and 6-BA (0, 50, or 100 mg·L^−1^) (Table 3). The experiment was conducted in 3 plant growth chambers. There were 3 biological replicates (*n* = 3) in each treatment. The data were collected after a month. The fully-expanded young leaves and crowns were harvested and immediately put into liquid nitrogen, then stored in a −80 °C freezer until further use. The rest of the strawberry plants were moved to a glasshouse and the growth indexes of the runners were measured after 5 weeks of cultivation.

### 4.2. Measurement of the Chlorophyll Content 

The chlorophyll content was measured with the Plus Chlorophyll Meter (Spectrum technologies, Wales, UK).

### 4.3. Proteomic Analysis 

#### 4.3.1. Materials for the Proteomic Analysis

As the photoperiod is the most influential factor for runner induction based on our results, we conducted another experiment to produce runners for the proteomic analysis. Strawberry plants were grown in a closed walk-in growth chamber with 25/15 ℃ day/night temperatures. Half of the strawberry plants were grown with the LD (16 h) photoperiod to produce runners, while the others were grown with the short day (SD, 10 h) photoperiod, using a light with an intensity of 300 μmol·m^−2^·s^−1^ PPFD. The crowns of the strawberry plants with 3 runners grown in LD and those with 0 runners grown in SD were collected after 2 months, were immediately frozen in liquid nitrogen, and subsequently stored in a −80 ℃ refrigerator. 

#### 4.3.2. Protein Extraction

The total proteins in the strawberry crowns were extracted based on the methods described by Bianco et al. [64] with minor modifications. Samples weighing 0.5 g were finely ground in liquid nitrogen. The powder was transferred into a 15 mL centrifuge tube, and was suspended with a 5 mL extraction buffer (0.7 M sucrose, 0.1 M KCl, 0.5 M Tris–HCl, pH 7.5, 50 mM EDTA, 1% PVP, 2% w/v β-mercaptoethanol and 1 mM PSMF, 10 mM DTT). The tube was kept in ice for 30 min before an equal volume of chilled phenol saturated with a Tris-HCl buffer (pH = 7.5) was added, and then was vortexed for 15 min. After a 20-min centrifugation at 5000 *g* and 4 ℃, the upper phenolic phase was collected and mixed with 5 volumes of 100 mM ammonium acetate in methanol, then was kept at −20 °C overnight. The precipitated proteins were centrifuged for 20 min at 5000 *g* and 4 °C, and then rinsed once with a pre-cooling methanol and twice with chilled 80% acetone. The proteins were then air-dried at room temperature and dissolved in a 400 μL rehydration buffer (7 M urea, 2 M thiourea, 4% CHAPS, 2% IPG buffer at pH 3–10 nonlinear and 0.001% bromophenol blue). Finally, the protein concentration was determined with the Bradford reagent (Sigma-Aldrich, St. Louis, MO, USA) according to the manual, and bovine serum albumin (BSA) was used as the standard.

#### 4.3.3. Two-Dimensional Gel Electrophoresis and Image Analysis

For the first dimension, isoelectric focusing (IEF) of proteins was performed based on the method described by Gorg et al. [65] with some modifications. The immobilized pH gradient (IPG) strips (18 cm, pH 3–10 NL) were passively rehydrated with 500 μg of protein in a 360 μL rehydration buffer in the IPGbox (GE Healthcare, Little Chalfont, Buckinghamshire, UK) for 12 h. The focusing was performed in the Ettan IPGphor 3 (GE Healthcare, UK) at 20 °C with 50 mA per strip under the following conditions: 30 V for 2 h, 200 V for 1 h, 500 V for 1 h, 3000 V for 1 h, gradient from 3000 V to 8000 V within 30 min, and 8000 V for 3 h. After the first dimensional IEF, the IPG strips were equilibrated according to the method of Gorg et al. [65]. The second dimensional electrophoresis was carried out in 12% SDS-PAGE, then the gels were stained with Coomassie brilliant blue (CBB) according to R-250/G-250 = 4:1. The images were taken with a high-resolution scanner (Epson, Long Beach, USA). The abundant difference (2-fold, *p* < 0.05) of the proteins in the samples were calculated with the PDQuest Advanced 2-D Analysis software (version 8.01, Bio-Rad Laboratories, Hercules, CA, USA) based on Student’s *t*-test.

#### 4.3.4. In-Gel Digestion and MALDI-TOF/MS

The differentially expressed proteins were excised from PAGE gel with pipette tips, and the in-gel digestion was performed according to the method of Shevchenko et al. [66]. The peptide solution was then spotted onto the MALDI-TOF MS target plate. The MALDI-MS analysis was performed using an ABI 4800 Plus TOF/TOF mass spectrometer (Applied Biosystems, Framingham, MA, USA). The running condition was set to 200 Hz ND: 355 nm YAG laser operations, and the 10 most and least intense ions per MALDI spot with signal/noise ratios >25 were selected for the subsequent MS/MS analysis in 1 kV mode with 800–1000 consecutive laser exposure. The MS/MS spectra data were searched against the NCBInr database and Protein Pilot v.3.0 software (AB Sciex, Framingham, MA, USA) with the MASCOT search engine (ver. 2.3.02, Matrix Science, London, UK) at 50 ppm of mass tolerance. Oxidation of methionines and carbamidomethylation of cysteines were allowed for the MS/MS spectra search in the databases. Individual peptide ion scores were searched using a statistically significant threshold value of *p* = 0.05.

#### 4.3.5. Protein Functional Classification

The identified proteins were classified into different categories of biological processes according to gene ontology [67].

### 4.4. Measurements of the Soluble Sugar and Starch Contents 

The soluble sugar and starch contents were determined by the anthrone–sulfuric acid colorimetry [68]. An amount of 0.3 g of leaf or crown samples were finely ground in liquid nitrogen and then extracted in 25 mL of distilled water for 30 min at 100 °C. Samples were centrifuged at 6500 rpm for 10 min before the supernatant was collected for the soluble sugar content assays. The residues of the leaves were suspended in 20 mL of distilled water and 2 mL of 9.2 M perchloric acid. The mixture was then placed in boiling water for 15 min, and after centrifugation, the supernatant was collected for the starch content assay. An amount of 0.2 mL of soluble sugar was transferred to 1.8 mL of distilled water, and 0.5 mL of starch was transferred to 1.5 mL of distilled water. An amount of 0.5 mL of 2% anthrone and 5 mL of concentrated sulfuric acid were then added to the soluble sugar or starch solutions. After the solutions were incubated in boiling water for 10 min, the absorbance of the mixed solution was measured at 630 nm for soluble sugar and 485 nm for starch with a UV-spectrophotometer (Libra S22, Biochrom Ltd., Cambridge, UK). The soluble sugar and starch contents were calculated based on the standard curve.

### 4.5. Measurements of the Soluble Sugar and Starch Contents 

The physiological parameters were obtained in a complete randomized design with three replicates, and are presented as the mean ± the standard deviation (SD). The data collected were subjected to an analysis of variance (ANOVA) followed by Duncan′s multiple range test at *p* < 0.05 with the SAS (Statistical Analysis System, V. 6.2, Cary, NC, USA) program. Furthermore, we conducted a range analysis to test the optimal level for runner induction. The equation used was R = max{*K_i_^X^*} − min{*K_i_^X^*}, where *K_i_^X^* is the average of the following variables: photoperiod, temperature, GA_3_ and 6-BA, at the 1, 2, and 3 levels. *X,* represents the photoperiod, temperature, GA_3_, or 6-BA and *i* represents the levels of 1, 2, or 3 [69].

## 5. Conclusions

We compared the effects of the photoperiod, temperature, and GA_3_ and 6-BA concentrations on runner induction in strawberry, and we found that the photoperiod was the most influential factor for runner induction. The expected optimal combination for runner formation was a 16-h photoperiod combined with 25/15 °C day/night temperatures, and 50 mg·L^−1^ of 6-BA. A proteomic analysis revealed that proteins related to carbohydrate metabolism and photosynthesis were the most prominent groups under the LD condition, indicating that more carbohydrates were produced for runner induction. Moreover, we demonstrated that the soluble sugar content was positively correlated with the number of runners, which suggested that sugar may play an important role in runner formation in strawberry.

## Figures and Tables

**Table 1:** Range analysis of L_9_ (3)^4^ test results for the number of runners.

Variable	Temperature	Photoperiod	GA_3_	BA
*K*_1_	6.00	2.67	18.00	5.00
*K*_2_	14.00	10.33	7.33	15.00
*K*_3_	10.67	17.67	5.33	10.67
R	8.00	15.00	12.67	10.00
Optimal level	25/15 °C	16/8 h	0 mg·L^−1^	50 mg·L^−1^

*K_i_* is the average of the following variables: photoperiod, temperature, GA_3_ and 6-BA, at the 1, 2, and 3 levels. R = max *Ki* – min *Ki*. The greatest values for K and R are expressed in bold.

**Table 2:** Proteins identified by the MALDI-TOF MS in strawberry.

Spot No.	Accession No.	Species	Protein Name	Score ^a^	Theor./Exp. M_r_ (kDa) ^b^	Theor./Exp. p*I* ^c^	SC (%) ^d^
Carbohydrate Metabolism							
3	gi|470120564	*Fragaria vesca*	Malate dehydrogenase, cytoplasmic-like	59	36.0/40.7	6.01/6.35	23
8	gi|470136472	*Fragaria vesca*	Glucose-6-phosphate 1-Dehydrogenase, cytoplasmic isoform 2-like	184	58.8/81.7	5.93/6.07	36
9	gi|470109046	*Fragaria vesca*	Sucrose synthase 2-like	98	92.8/90.8	5.94/6.01	23
20	gi|470148237	*Fragaria vesca*	Glucan endo-1,3-beta-glucosidase	157	16/31.9	5.33/5.58	32
25	gi|470114187	*Fragaria vesca*	Putative lactoylglutathione lyase-like isoform 2	114	32.6/31.0	5.28/5.33	33
Proteometabolism							
6	gi|470132774	*Fragaria vesca*	Probable protein disulfide-isomerase A6-like	81	40.1/47.2	6.12/6.02	30
7	gi|470136937	*Fragaria vesca*	Probable mitochondrial-processing peptidase subunit beta-like	219	58.5/60.7	6.42/6.07	29
12	gi|470114303	*Fragaria vesca*	Fumarylacetoacetase-like	69	45.8/45.3	5.57/5.89	32
23	gi|470142843	*Fragaria vesca*	26S protease regulatory subunit 6B homolog	99	46.2/55.6	5.48/5.54	26
Photosynthesis							
13	gi|7008071	*Baccharis halimifolia*	Ribulose-1,5-bisphosphate carboxylase/oxygenase large subunit	68	54.5/57.5	6.29/5.92	20
14	NES2_FRAAN	*Fragaria ananassa*	(3S,6E)-nerolidol synthase 2, chloroplastic/mitochondrial OS	40	66.6/88.6	6.27/5.99	20
19	gi|470122943	*Fragaria vesca*	Chaperone protein ClpC, Chloroplastic-like	93	102.4/94.1	6.59/5.63	27
24	gi|237637006	*Linum tenue*	Ribulose-1,5-bisphosphate carboxylase/oxygenase large subunit	94	51.2/89.2	6.23/5.46	17
28	gi|470107388	*Fragaria vesca*	Chaperonin CPN60-2, mitochondrial-like	89	61.8/69.9	5.89/5.41	28
Respiration							
10	gi|470109651	*Fragaria vesca*	Coenzyme Q-binding protein COQ10 homolog, mitochondrial-like	45	28.6/31.5	8.85/5.92	24
Signaling Proteins							
2	gi|470103566	*Fragaria vesca*	Annexin D1-like	263	36.4/31.5	6.44/6.54	44
5	gi|470114874	*Fragaria vesca*	Transcription factor bHLH135-like	53	10.4/12.2	5.07/6.02	44
16	gi|571467514	*Glycine max*	Probable calcium-binding protein CML45-like	70	19.5/32.3	4.64/5.74	40
Stress-Related Proteins							
1	gi|470129560	*Fragaria vesca*	Catalase-like	87	57.1/59.7	6.58/6.74	33
4	gi|470126676	*Fragaria vesca*	Chaperone protein ClpB1-like	85	101.8/100.5	5.91/6.21	21
31	gi|470121677	*Fragaria vesca*	Major allergen Pru ar 1-like	36	17.7/22.9	5.39/5.51	44
32	gi|470106694	*Fragaria vesca*	Pathogenesis-related protein 1C-like	70	18.1/34.2	5.1/5.05	24
General Metabolism-Related Proteins							
11	gi|470134340	*Fragaria vesca*	Monodehydroascorbate reductase-like	72	47.2/48.6	6.22/5.98	23
17	gi|51493451	*Fragaria ananassa*	Flavanone 3-hydroxylase	307	41.4/43.1	5.61/5.72	53
29	gi|470144168	*Fragaria vesca*	Chalcone-flavonone isomerase-like	95	23.6/25.4	4.86/5.16	46

^a^ score, MASCOT score of protein hit. ^b^ Theoretical and experimental molecular mass (Mr) calculated by MASCOT peptide mass fingerprint and protein gel images, respectively. ^c^ Isoelectric point (pI) of spots identified by MASCOT and protein gel images. ^d^ SC, sequence coverage (%).

**Table 3:** L_9_ (3)^4^ orthogonal design used in this study.

Treatment	Temperature (A, D/N, °C)		Photoperiod (B, D/N, h)		GA_3_ (C, mg·L^−1^)		6-BA (D, mg·L^−1^)	
T1	A_1_	23/17	B_1_	8/16	C_1_	0	D_1_	0
T2	A_1_	23/17	B_2_	12/12	C_2_	50	D_2_	50
T3	A_1_	23/17	B_3_	16/8	C_3_	100	D_3_	100
T4	A_2_	25/15	B_1_	8/16	C_2_	50	D_3_	100
T5	A_2_	25/15	B_2_	12/12	C_3_	100	D_1_	0
T6	A_2_	25/15	B_3_	16/8	C_1_	0	D_2_	50
T7	A_3_	27/13	B_1_	8/16	C_3_	100	D_2_	50
T8	A_3_	27/13	B_2_	12/12	C_1_	0	D_3_	100
T9	A_3_	27/13	B_3_	16/8	C_2_	50	D_1_	0

A, B, C, and D represent the temperature, photoperiod, GA_3_, and 6-BA, respectively; D/N means day/night.

## Supplementary Materials

Supplementary materials can be found at https://www.mdpi.com/1422-0067/21/14/4917/s1.
