# Supplementary material for: A Long-Day Photoperiod and 6-Benzyladenine Promote Runner Formation through Upregulation of Soluble Sugar Content in Strawberry
Source: Int J Mol Sci. 2020 Jul 12;21(14):4917. doi: 10.3390/ijms21144917 (PMC7403970; doi:10.3390/ijms21144917)
Supplement: Supplementary file 1 [file ijms-21-04917-s001.pdf]

Table 1. Range analysis of the $L_9\,(3)^4$ test results for the plant height.

Variable	Temperature	Photoperiod	GA_3	BA
K_1	**26.92**	**27.31**	20.43	26.23
K_2	25.02	26.27	28.27	25.48
K_3	26.71	25.08	**29.96**	**26.94**
R	1.90	2.23	**9.53**	1.47
Optimal level	23/17°C	8 h	100 mg·L^{-1}	100 mg·L^{-1}

Table 2. Range analysis of the $L_9\,(3)^4$ test results for the petiole length.

Variable	Temperature	Photoperiod	GA_3	BA
K_1	**17.94**	**18.49**	12.96	17.36
K_2	15.98	17.08	18.65	14.48
K_3	17.32	15.68	**19.63**	**17.41**
R	1.96	2.81	**6.67**	2.93
Optimal level	23/17°C	8 h	100 mg·L^{-1}	100 mg·L^{-1}

Table 3. Range analysis of the $L_9\,(3)^4$ test results for the leaf length.

Variable	Temperature	Photoperiod	GA_3	BA
K_1	8.98	8.69	7.42	8.91
K_2	8.94	9.19	9.54	8.94
K_3	**9.29**	**9.34**	**10.26**	**9.37**
R	0.35	0.65	**2.84**	0.46
Optimal level	23/17°C	8 h	100 mg·L^{-1}	100 mg·L^{-1}

Table 4. Range analysis of the $L_9\,(3)^4$ test results for the leaf width.

Variable	Temperature	Photoperiod	GA_3	BA
K_1	**7.07**	6.27	6.07	6.55
K_2	6.62	6.86	6.79	6.63
K_3	6.58	**7.15**	**7.41**	**7.09**
R	0.49	0.88	**1.34**	0.54
Optimal level	23/17°C	16 h	100 mg·L^{-1}	100 mg·L^{-1}

Table 5. Range analysis of the $L_9\,(3)^4$ test results for the runner length.

Variable	Temperature	Photoperiod	GA_3	BA
K_1	**27.89**	19.01	11.27	12.11
K_2	20.04	20.92	19.79	26.23
K_3	21.67	**29.68**	**38.55**	**31.26**

R	7.84	10.67	**27.29**	19.16
Optimal level	23/17°C	12 h	100 mg·L^{-1}	100 mg·L^{-1}

Table 6. Range analysis of the $L_9(3)^4$ test results for the chlorophyll content.

Variable	Temperature	Photoperiod	GA_3	BA
K_1	35.43	36.54	**43.42**	37.38
K_2	37.04	38.64	32.99	**38.44**
K_3	**41.68**	**38.97**	37.74	38.33
R	6.26	2.43	**11.42**	1.06
Optimal level	23/17°C	8 h	100 mg·L^{-1}	100 mg·L^{-1}

Table 7. Range analysis of the $L_9(3)^4$ test results for the soluble sugar content.

Variable	Temperature	Photoperiod	GA_3	BA
K_1	5.19	4.09	**7.45**	4.76
K_2	**5.37**	**6.16**	4.28	**5.91**
K_3	5.22	5.53	4.05	5.11
R	0.18	2.07	**3.40**	1.16
Optimal level	25/15°C	12 h	0 mg·L^{-1}	50 mg·L^{-1}

Table 8. Range analysis of the $L_9(3)^4$ test results for the starch content.

Variable	Temperature	Photoperiod	GA_3	BA
K_1	1.74	1.63	**2.08**	1.60
K_2	1.73	**1.85**	1.64	**1.95**
K_3	**1.84**	1.83	1.59	1.76
R	0.12	0.23	**0.49**	0.35
Optimal level	27/13°C	12 h	0 mg·L^{-1}	50 mg·L^{-1}

The greatest values for K and R are expressed in bold. The equation used was $R = \max\{K_i^X\} - \min\{K_i^X\}$, where K_i^X is the average of the following variables: photoperiod, temperature, GA_3 and 6-BA, at the 1, 2, and 3 levels; X represents the photoperiod, temperature, GA_3, or 6-BA; and i is 1, 2, or 3.

Table 9. GO classification of the DEPs identified in this study.

Spot no.	protein name	GO annotation		
		Biological Process	Molecular Function	Cellular Component
1	Catalase-like	Hydrogen peroxide catabolic process; oxidation-reduction process	Metal ion binding; oxidation-reduction process	Peroxisome
2	Annexin D1-like	acetyl-CoA metabolic process; polysaccharide transport; brassinosteroid biosynthetic process;	Calcium ion binding; calcium-dependent phospholipid binding;	Cytosol; thylakoid; cell surface; apoplast
3	Malate dehydrogenase, cytoplasmic-like	Response to cold	L-malate dehydrogenase activity	Mitochondrion; vacuolar membrane; chloroplast stroma; chloroplast envelope; apoplast
4	Chaperone protein ClpB1-like	Protein folding; response to high light intensity; heat acclimation; response to endoplasmic reticulum stress; response to hydrogen peroxide; protein unfolding; positive regulation of translation	Protein binding; ATP binding; ATPase activity	Chloroplast stroma; chloroplast envelope
5	Transcription factor bHLH135-like		Protein dimerization activity	——
6	Probable protein disulfide-isomerase A6-like	Oxidation-reduction process	Naringenin 3-dioxygenase activity	——
7	Probable mitochondrial-processing peptidase subunit beta-like	Proteolysis	Metalloendopeptidase activity; metal ion binding	——
8	Glucose-6-phosphate 1-Dehydrogenase, cytoplasmic isoform 2-like	Pentose-phosphate shunt, oxidative branch	Glucose-6-phosphate dehydrogenase activity	Cytosol
9	Sucrose synthase 2-like	Cytokinesis by cell plate formation; response to hypoxia; starch metabolic process; sucrose biosynthetic process; N-terminal protein myristoylation; cell-cell signaling; meristem initiation; vegetative phase change; meristem maintenance;	Sucrose synthase activity	Nucleus; cytosol; chloroplast
10	Coenzyme Q-binding protein COQ10 homolog, mitochondrial-like	Aromatic amino acid family metabolic process; oxidation-reduction process	4-hydroxyphenylpyruvate dioxygenase activity	——

11	Monodehydroascorbate reductase-like	Glucosinolate biosynthetic process; hydrogen peroxide catabolic process; response to cadmium ion	Monodehydroascorbate reductase (NADH) activity	Peroxisomal membrane; chloroplast envelope;
12	Fumarylacetoacetase-like	Tyrosine catabolic process; cell death; chlorophyll catabolic process; homogentisate catabolic process	Fumarylacetoacetase activity	Cytosol
13	Ribulose-1,5-bisphosphate carboxylase/oxygenase large subunit	Photorespiration; oxidation-reduction process	Magnesium ion binding; monooxygenase activity; ribulose-bisphosphate carboxylase activity	Chloroplast
14	(3S,6E)-nerolidol synthase 2, chloroplastic/mitochondrial OS	Terpenoid biosynthetic process	Magnesium ion binding; terpene synthase activity;	Mitochondrion; chloroplast
16	Probable calcium-binding protein CML45-like	——	——	——
17	Flavanone 3-hydroxylase	Oxidation-reduction process	Naringenin 3-dioxygenase activity	——
19	Chaperone protein ClpC, Chloroplastic-like	Proteolysis	ATP binding; peptidase activity; nucleoside-triphosphatase activity	Chloroplast
20	Glucan endo-1,3-beta-glucosidase	Carbohydrate metabolic process	Hydrolase activity, hydrolyzing O-glycosyl compounds	——
23	26S protease regulatory subunit 6B homolog	——	——	——
24	Ribulose-1,5-bisphosphate carboxylase/oxygenase large subunit	Carbon fixation; photosynthesis;	Magnesium ion binding; ribulose-bisphosphate carboxylase activity	Chloroplast
25	Putative lactoylglutathione lyase-like isoform 2	Gluconeogenesis; glycolytic process; response to salt stress	Lactoylglutathione lyase activity; calmodulin binding; metal ion binding	Cytosol
28	Chaperonin CPN60-2, mitochondrial-like	Response to stress; protein refolding	ATP binding	Mitochondrion
29	Chalcone-flavonone isomerase-like	Response to sucrose; response to UV-B; response to karrikin	——	Chloroplast
31	Major allergen Pru ar 1-like	Defense response; response to biotic stimulus	——	
32	Pathogenesis-related protein 1C-like	——	——	Extracellular region

Table 10. KEGG classification of the DEPs identified in this study.

#Pathway	ko_ID	Unigene	gene	Unigene_all	Spot no.
Ascorbate and aldarate metabolism	ko00053	1	1	10	11
Carbon fixation in photosynthetic organisms	ko00710	3	3	10	13;24;3
Carbon metabolism	ko01200	4	4	10	13;24;3;8
Citrate cycle (TCA cycle)	ko00020	1	1	10	3
Phosphatidylinositol signaling system	ko04070	1	1	10	16
Glutathione metabolism	ko00480	1	1	10	8
Tyrosine metabolism	ko00350	1	1	10	12
Starch and sucrose metabolism	ko00500	1	1	10	9
Glyoxylate and dicarboxylate metabolism	ko00630	3	3	10	13;24;3
RNA degradation	ko03018	1	1	10	28
Protein processing in endoplasmic reticulum	ko04141	1	1	10	6
Plant-pathogen interaction	ko04626	1	1	10	16
Pentose phosphate pathway	ko00030	1	1	10	8
Pyruvate metabolism	ko00620	1	1	10	3
Cysteine and methionine metabolism	ko00270	1	1	10	3

Table 11. COG classification of DEPs identified in this study.

#Gene name	Portein ID in COG	E_value	Identity	Score	COG id	COG class defination	Function code	Functional categories	Function class defination
1	gi|449088034|ref|YP _007420475.1|	3.00E-171	504	504	COG0753	Catalase	[P]	Metabolism	Inorganic ion transport and metabolism
3	gi|121997621|ref|YP _001002408.1|	5.00E-116	352	352	COG0039	Malate/lactate dehydrogenase	[C]	Metabolism	Energy production and conversion

4	gi|269926323|ref|YP_003322946.1|	0	870	870	COG0542	ATP-dependent Clp protease ATP-binding subunit ClpA	[O]	Cellular processes and signaling	Posttranslational modification, protein turnover, chaperones
6	gi|20807685|ref|NP_622856.1|	2.00E-26	112	112	COG3118	Negative regulator of GroEL	[O]	Cellular processes and signaling	Posttranslational modification, protein turnover, chaperones
7	gi|392382816|ref|YP_005032013.1|	3.00E-94	305	305	COG0612	Predicted Zn-dependent peptidase	[R]	Poorly characterized	General function prediction only
8	gi|383452925|ref|YP_005366914.1|	3.00E-123	384	384	COG0364	Glucose-6-phosphate 1-dehydrogenase	[G]	Metabolism	Carbohydrate transport and metabolism
9	gi|292493898|ref|YP_003529337.1|	0	880	880	COG0438	Glycosyltransferase involved in cell wall bisynthesis	[M]	Cellular processes and signaling	Cell wall/membrane/envelope biogenesis
10	gi|414343662|ref|YP_006985183.1|	2.00E-25	107	107	COG2867	Ribosome association toxin PasT (RatA) of the RatAB toxin-antitoxin module	[J]	Information storage and processing	Translation, ribosomal structure and biogenesis
11	gi|116622785|ref|YP_824941.1|	9.00E-63	219	219	COG0446	NADPH-dependent 2	[I]	Metabolism	Lipid transport and metabolism
13	gi|434391829|ref|YP_007126776.1|	0	812	812	COG1850	Ribulose 1	[G]	Metabolism	Carbohydrate transport and metabolism
17	gi|470176812|ref|YP_007562856.1|	8.00E-14	77.8	77.8	COG3491	Isopenicillin N synthase and related dioxygenases	[Q]	Metabolism	Secondary metabolites biosynthesis, transport and catabolism
19	gi|158335106|ref|YP_001516278.1|	0	1189	1189	COG0542	ATP-dependent Clp protease ATP-binding subunit ClpA	[O]	Cellular processes and signaling	Posttranslational modification, protein turnover, chaperones

23	gi|11499558|ref|NP_070800.1|	7.00E-122	374	374	COG1222	ATP-dependent 26S proteasome regulatory subunit	[O]	Cellular processes and signaling	Posttranslational modification, protein turnover, chaperones
24	gi|434391829|ref|YP_007126776.1|	0	798	798	COG1850	Ribulose 1	[G]	Metabolism	Carbohydrate transport and metabolism
25	gi|17545239|ref|NP_518641.1|	1.00E-49	171	171	COG0346	Catechol 2	[Q]	Metabolism	Secondary metabolites biosynthesis, transport and catabolism
28	gi|389877554|ref|YP_006371119.1|	0	690	690	COG0459	Chaperonin GroEL (HSP60 family)	[O]	Cellular processes and signaling	Posttranslational modification, protein turnover, chaperones
32	gi|427726216|ref|YP_007073493.1|	5.00E-31	123	123	COG2340	Uncharacterized conserved protein YkwD	[S]	Poorly characterized	Function unknown
